# Influence of Build Platform Position on the Trueness and Precision of LCD 3D-Printed Orthognathic Surgical Splints

**DOI:** 10.3390/dj14030145

**Published:** 2026-03-05

**Authors:** Siripatra Patchanee, Pokpong Amornvit, Aunchisa Sreatsiripakdee, Chantawat Tanapirom, Narissaporn Chaiprakit

**Affiliations:** 1 Division of Orthodontics, Faculty of Dentistry, Thammasat University, Pathum Thani 12120, Thailand; siripatra.p@hotmail.com; 2 PPFACEDESIGN, The S Clinic, Bangkok 10120, Thailand; pokpong_am@yahoo.com; 3 Undergraduate Program in Doctor of Dental Surgery, Faculty of Dentistry, Thammasat University, Khlong Luang 12120, Thailand; aunchisa.sre@gmail.com (A.S.); earthchantawat@gmail.com (C.T.); 4 Division of Oral and Maxillofacial Surgery, Faculty of Dentistry, Thammasat University, Khlong Luang 12120, Thailand

**Keywords:** orthognathic surgery, splint, CAD/CAM, 3D printer

## Abstract

**Background/Objectives**: Among vat polymerization technologies, liquid-crystal display (LCD) 3D printing has gained popularity in dentistry because of its affordability and acceptable resolution. However, the factors influencing the dimensional accuracy of LCD-printed surgical splints, particularly build platform position, remain insufficiently investigated. This study aimed to evaluate the influence of build platform position on the trueness and precision of orthognathic surgical splints fabricated using LCD 3D printing technology. **Methods**: Thirty-six surgical splints were printed from a master digital file using an LCD 3D printer. All surgical splints were printed with a 90-degree layer orientation to the building platform. The layer thickness was set at 100 μm. The surgical splints were divided into three groups according to their printing position on the building platform: middle (M), left (Lt), and right (Rt). Each 3D-printed surgical splint was sprayed with an opaque scanning spray and then rescanned to create digital files for testing. A surface-based superimposition and deviation analysis was performed using specialized 3D software to evaluate accuracy of surgical splints. Root mean square error (RMSE) values were statistically analyzed. **Results**: There were no statistically significant differences in trueness among the middle, left, and right printing positions on the build platform (*p* > 0.05). In contrast, printing position significantly affected precision, with surgical splints printed at the center of the build platform demonstrating significantly lower RMSE values compared with those printed at the left and right positions (*p* < 0.001). In addition, no significant difference in precision was detected between the left and right positions. **Conclusions**: The printing position on the build platform significantly influences the precision of orthognathic surgical splints fabricated using LCD 3D printing technology. Splints printed at the central region of the build platform exhibited the highest precision, whereas trueness was not significantly affected by printing position. These findings suggest that preferential placement of surgical splints at the center of the build platform may improve fabrication consistency and predictability in digital orthognathic surgery workflows.

## 1. Introduction

Orthognathic surgery (OGS) procedures are primarily performed for the definitive correction of skeletal Class II and III deformities, mandibular deviation, and maxillofacial asymmetry. For many years, several studies have highlighted the importance of three-dimensional (3D) planning in orthognathic surgery. Accurate treatment planning is essential in orthognathic surgery to achieve optimal esthetic and occlusion outcomes. Recent advances in 3D imaging with cone beam computed tomography (CBCT) have enabled computer-assisted orthognathic surgery, which has introduced detailed visualization of the craniofacial complex and enhanced surgical planning to improve the predictability of outcomes [1]. Preoperative planning requires data collection to establish an accurate diagnosis of the dentoskeletal deformity and a treatment plan that can be reproduced reliably in the operating room. Advances in 3D imaging technology have led to the development of computerized tools for preoperative planning and the fabrication of orthognathic surgical splints [2]. In orthognathic surgery, surgical splints are critical tools for repositioning the maxilla and mandible. However, traditional laboratory methods for fabricating surgical splints have been associated with certain limitations [1].

The conventional production of surgical splints involves the use of plaster models, a facebow, and an articulator. However, these manual procedures are time-consuming and prone to uncontrollable errors and inter-laboratory variability. The shift from 2D to 3D imaging provides clinicians and patients with enhanced information for diagnosis, treatment planning, and simulation of post-treatment outcomes [3]. Intraoral scanners enable dentists to record patients’ dentition without the discomfort associated with traditional impressions and reduce the shrinkage errors of alginate impressions and stone models. The use of intraoral scanners produces 3D images that can be digitally processed and printed [4]. Compared with traditional methods, digitally fabricated surgical splints offer high precision and repeatability along with improved power and competency [5]. Widely applied 3D printing technologies in dentistry are stereolithography (SLA), digital light processing (DLP), and liquid-crystal display (LCD) systems. All these additive resin technologies utilize vat polymerization techniques. These 3D printers create objects by curing liquid photopolymer resin using different light sources [6].

LCD 3D printing operates under the same vat polymerization mechanism as stereolithography (SLA) and digital light processing (DLP) systems. In this technology, a liquid-crystal display panel functions as the light-masking component that selectively cures the photopolymer resin. The emitted light is transmitted directly and uniformly toward the build platform without the use of projection lenses. Because the illumination is delivered in a parallel manner rather than through optical projection, distortion related to pixel scaling is minimized in LCD-based printers. Compared with SLA technology, LCD 3D printers offer faster printing speeds. LCD printers offer printing rates similar to DLP technology, while typically providing a more economical option due to reduced material and manufacturing costs. Additional benefits of LCD-based 3D printing include the capability to fabricate transparent components as well as large models with high spatial resolution. These advantages have contributed to the increasing availability of LCD 3D printers on the dental market [4].

In accordance with ISO 5725-1(International Organization for Standardization: Geneva, Switzerland; 1994.), accuracy comprises two components: precision and trueness. Precision refers to the reproducibility of repeated measurements under defined conditions, whereas trueness indicates the degree of agreement between the mean measured value and the true or reference dimension. Recent scoping reviews highlight the expanding literature on 3D-printed aligners, noting both in-office production feasibility and growing clinical interest in their accuracy and fit. These developments underscore the broader relevance of printing accuracy research, as factors like build platform positioning may similarly impact other orthodontic applications [7].

One of the principal distinctions between DLP and LCD-based vat polymerization systems is the variation in light output intensity. Because photopolymerization kinetics are governed by the delivered light energy, resins optimized for DLP projection may require modification either through increased initiator concentration or longer irradiation periods which can achieve comparable curing performance in LCD printers [8].

LCD 3D printing systems are generally considered an economical option and provide resolution that is suitable for many dental applications. Nevertheless, these devices are associated with certain technical limitations. The lifespan of LCD panels is relatively limited, necessitating periodic replacement. Additionally, only a small proportion of emitted light is transmitted through the LCD mask to the resin surface, as a substantial amount of light energy is absorbed by the screen itself. This reduced light transmission may affect curing efficiency. Furthermore, minor light leakage can occur during printing, potentially leading to unintended polymerization within the resin vat and requiring routine maintenance and cleaning. Currently, LCD 3D photocuring machines are used in dentistry, jewelry making, toy manufacturing, and other industries [8].

Several factors have been reported to influence the accuracy of vat polymerization-based 3D printing systems, including printing orientation, layer thickness, light intensity distribution, exposure time, build platform position [9], post-processing conditions [10], scanner-related variables, support structure design, and resin composition [11]. Variations in these parameters may affect the polymerization shrinkage, dimensional stability, and repeatability of printed splints, models, and orthodontic aids [12]. However, while the effects of orientation and post-processing have been investigated extensively, the influence of build platform position remains insufficiently clarified, particularly in LCD-based systems.

The statistical null hypothesis (H_0_) was that there would be no statistically significant differences in RMSE values among orthognathic surgical splints fabricated at different build platform positions (left, middle, and right) using LCD 3D printing technology.

## 2. Materials and Methods

**Figure 1:**
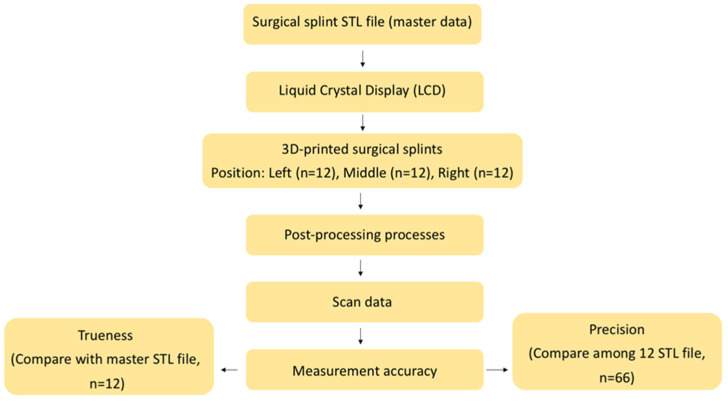
Conceptual framework of our research protocol.

This was an in vitro experimental study. A priori sample size calculation was conducted using G*Power software (version 3.1.9.6; Kiel University). Input parameters were 0.5 (effect size), 0.05 (alpha), 0.8 (power), and 3 (number of groups). The effect size was conservatively set at 0.5 to avoid overestimation of the expected difference, although previous in vitro studies have reported effect sizes of approximately 0.7 [9]. Twelve 3D-printed surgical stents were required per group, resulting in a total of 36 stents printed in this study. The conceptual framework of this study is demonstrated in Figure 1.

### 2.1. The 3D-Printing Protocol

A method modified from American National Standards Institute/American Dental Association (ANSI/ADA) Standard No. 132 for scanning accuracy was used in this study [13]. The master data for the surgical splint were obtained from previous research [10] and converted into a master standard tessellation language (STL) file, which was used as the original designed model in this study. The master STL file was inspected for mesh integrity and was standardized prior to printing to ensure uniform geometry across all specimens. To ensure consistent printing conditions, the build platform underwent leveling and calibration procedures in compliance with the manufacturer’s instructions prior to each print. The same exposure parameters, layer curing time, and bottom layer settings were applied for all specimens to eliminate variability related to printer configuration. All splints were printed in a single batch per group to minimize inter-batch variability. The STL file was imported into the 3D printing software (ALPHA AI Slicing Software) and printed using CURO Guide resin and an LCD 3D printer (Sol; Ackuretta Technologies, Taipei, Taiwan) (Table 1).

**Figure 2:**
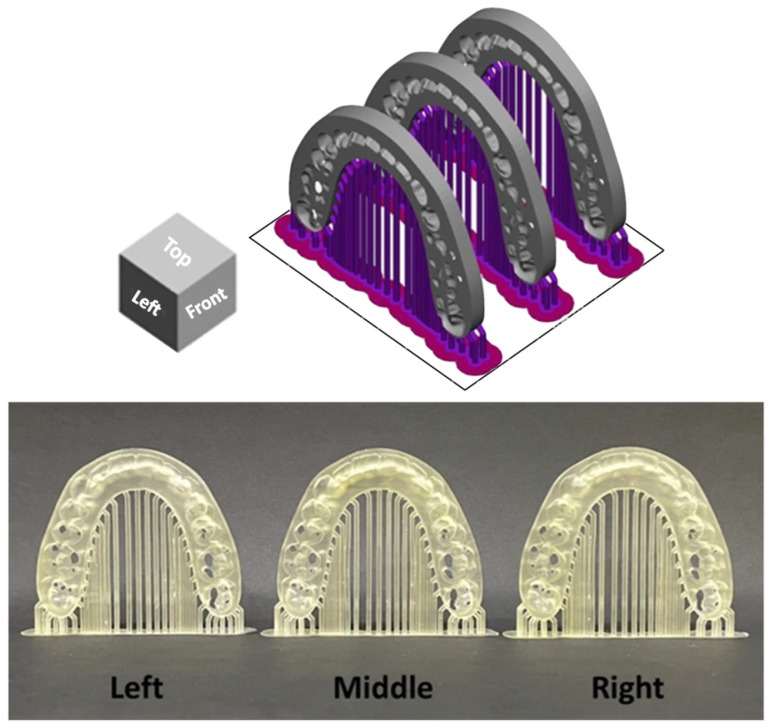
Arrangement of orthognathic surgical splints at different build platform positions. The upper panel illustrates the digital layout of the splints within the slicing software, showing the predefined left, middle, and right positions on the build platform. The lower panel presents the corresponding surgical splints after the printing process was completed.

The surgical splints were printed at a 90-degree orientation perpendicular to the build plate with each layer thickness of 100 μm. The surgical splints were categorized into three groups by printing position on the build platform: middle (M), left (Lt), and right (Rt) on the building platform (Figure 2) [14]. A total of 36 models were printed. After printing, the models were cleaned for 6 min with 99.9% isopropyl alcohol using CLEANI (Ackuretta Technologies, Taipei, Taiwan) to remove excess resin, then cured for 6 min in a post-curing oven using UV CURIE (Ackuretta Technologies, Taipei, Taiwan) according to the manufacturer’s instructions.

### 2.2. Surface-Based Superimposition for Data Analysis

The 3D-printed surgical splints were lightly coated with anti-reflective spray (Renfert Scan Spray, Renfert USA, Illinois, IL, USA). A consistent scanning protocol and scanning path were applied for all specimens, and anti-reflective spray was applied uniformly before scanning. All surgical splints were scanned using the same laboratory optical scanner (E3, 3Shape, Copenhagen, Denmark). The scan data were saved and exported as STL files (experimental data).

### 2.3. Accuracy Analysis

In accordance with ISO 5725-1 (International Organization for Standardization: Geneva, Switzerland; 1994.), accuracy was decomposed into trueness and precision components. Trueness was calculated by superimposing individual printed splints onto the master STL reference (12 datasets per position). Precision was derived from pairwise intra-group alignments among splints fabricated at the same platform location, yielding 66 comparisons per group.

### 2.4. Deviation Analysis (Calculation of Trueness and Precision)

The STL files of the superimposed models were imported into CloudCompare software (version 2.13.2; EDF, Paris, France) to calculate the distances between the superimposed surfaces. A best-fit alignment algorithm was selected prior to surface difference calculation to minimize global misalignment between datasets. Object alignment was performed using iterative closest point (ICP) registration in CloudCompare software. A standardized region of interest was selected for all specimens to ensure consistent surface comparison. Outlier filtering thresholds were kept constant across all comparisons to avoid bias in RMSE computation. The point-to-point surface deviations between the two aligned digital models were quantified and expressed as root mean square error (RMSE) values, which reflect the overall magnitude of deviation within a shared coordinate system. Depending on the selected reference dataset, the computed RMSE corresponded to either trueness (comparison with the master model) or precision (comparison among specimens fabricated at the same printing position) [15]. The calculated deviation datasets were exported as text files for further analysis. RMSE values (μm) were used as the primary metric for evaluating dimensional accuracy and were determined according to the following equation:

$$\mathrm{RMSE}\;=\;\frac{1}{\sqrt{n}}\;⦁\;\sqrt{\sum_{i=1}^{n}{\left(x_{1,i}-x_{2,i}\right)}^{2}}$$

where x_1,i_ represents measurement point i in the master data; x_2,i_ represents measurement point i in the experimental data; and *n* is the total number of points [8].

Deviation outcomes were additionally illustrated using three-dimensional color mapping to facilitate visual interpretation of surface discrepancies. A tolerance range of ±50 μm was defined as the nominal threshold, while ±100 μm was established as the critical limit. For trueness evaluation, the color maps depicted the mean deviation between each printed specimen and the master reference model. In precision analysis, the maps represented the average deviation among specimens fabricated at the same build platform position. Yellow-to-red areas indicate deviations exceeding the positive limit, while turquoise-to-dark blue areas indicate deviations below the negative limit. Acceptable deviations are represented by light green-to-green areas [15,16].

### 2.5. Statistical Analysis

All statistical procedures were performed using SPSS software (version 23; IBM Corp., Armonk, NY, USA). The assumption of normal distribution for RMSE values was verified using the Kolmogorov–Smirnov and Shapiro–Wilk tests. Intergroup comparisons of printing positions were conducted via one-way ANOVA, with Tukey’s honestly significant difference test applied for subsequent pairwise analyses when appropriate. A significance criterion of α = 0.05 was adopted for all tests.

## 3. Results

The normality of RMSE values for both trueness (Table 2) and precision (Table 3) was assessed. For trueness, RMSE values from all printing positions (left, middle, and right) showed no significant deviation from normal distribution (*p* > 0.05). Similarly, RMSE values for precision across all printing positions were normally distributed (*p* > 0.05). These results confirmed that the assumptions for subsequent parametric statistical analyses were satisfied.

**Figure 3:**
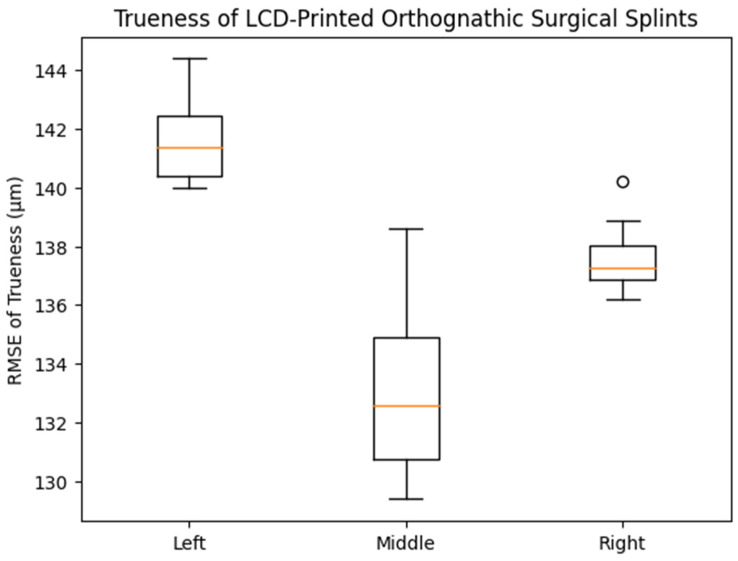
Box plot showing the distribution of trueness (RMSE, μm) of LCD-printed orthognathic surgical splints fabricated at different build platform positions (left, middle, and right). The central line within each box (orange line) represents the median value, while the box boundaries indicate the interquartile range (25th–75th percentile). The whiskers show the range of non-outlier values, and the small circles represent outliers. No statistically significant differences in trueness were observed among the groups (one-way ANOVA, *p* = 0.123).

For trueness, the mean RMSE values were 141.0 ± 2.15 μm (left), 134.6 ± 2.73 μm (middle), and 137.8 ± 1.30 μm (right), with no statistically significant differences among the groups (one-way ANOVA, *p* = 0.123). The 95% confidence intervals showed overlapping ranges across all positions. This finding is consistent with the distribution patterns observed in the box plot (Figure 3), which demonstrated comparable medians and interquartile ranges among the three printing positions.

**Figure 4:**
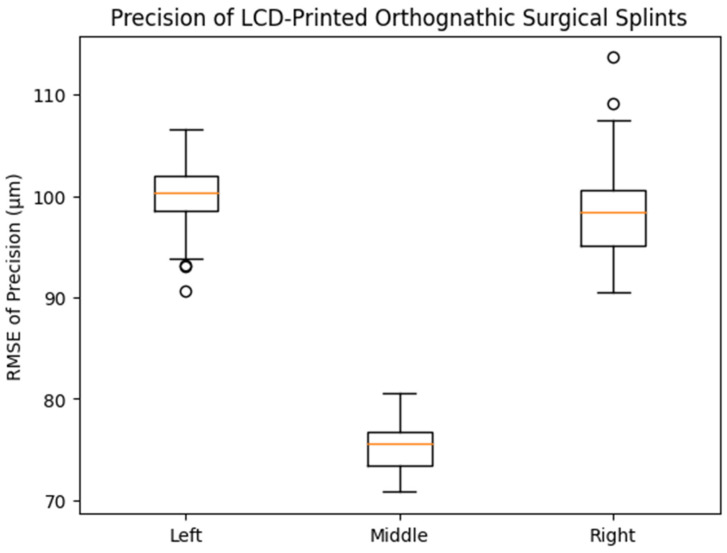
Box plot showing the distribution of precision (RMSE, μm) of LCD-printed orthognathic surgical splints fabricated at different printing positions on the build platform. The middle printing position demonstrated significantly lower RMSE values compared with the left and right positions (one-way ANOVA with Tukey HSD post hoc test, *p* < 0.001), whereas no significant difference was observed between the left and right positions.

In contrast, precision differed significantly according to printing position (*p* < 0.001). The middle printing position exhibited the lowest mean RMSE value (75.1 ± 2.23 μm), followed by the right (98.1 ± 4.04 μm) and left (100.6 ± 3.81 μm) positions. Post hoc analysis indicated statistically significant differences between the middle and both lateral positions, whereas no significant difference was observed between the left and right groups. As illustrated in the corresponding box plot (Figure 4), the middle group demonstrated a lower median and narrower interquartile range compared with the left and right groups, visually confirming the improved precision achieved at the central build platform position.

The results of the post hoc Tukey HSD analysis for trueness are presented in Table 4. Pairwise comparisons revealed no statistically significant differences in RMSE values among the left, middle, and right printing positions (*p* > 0.05 for all comparisons). These findings indicate that printing position on the build platform did not significantly affect the trueness of LCD-printed orthognathic surgical splints.

Subsequent Tukey HSD pairwise testing revealed that precision at the middle build platform position was significantly different from that at the left and right positions. In contrast, the comparison between the left and right groups did not reach statistical significance (Table 5).

**Figure 5:**
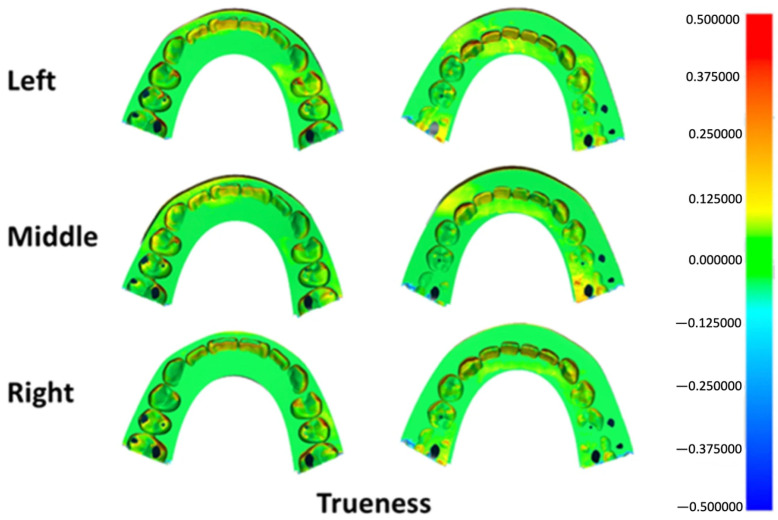
Three-dimensional color map illustrating trueness deviations across different build platform positions. Green shades represent areas within the acceptable tolerance range, warm colors (yellow to red) indicate positive surface deviations, and cool colors (light to dark blue) denote negative deviations.

**Figure 6:**
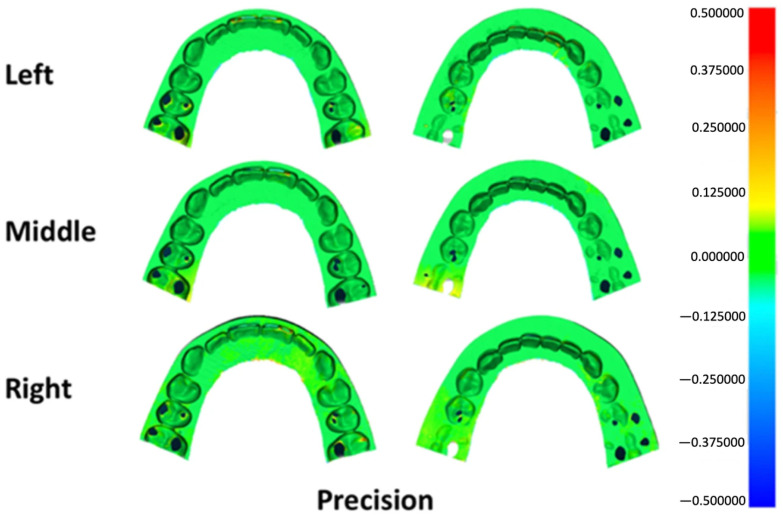
Three-dimensional color map illustrating precision deviations across different build platform positions. Green shades represent areas within the acceptable tolerance range, warm colors (yellow to red) indicate positive surface deviations, and cool colors (light to dark blue) denote negative deviations.

Surface deviation mapping for trueness identified localized positive discrepancies at anatomically prominent regions, including the incisal edges, canine cusp tip, premolar cusp tips, and the mesial surface of the first molar across all groups (Figure 5). Precision maps revealed small bidirectional deviations at the incisal region in each group, along with localized positive deviations at the mesiobuccal cusp of the first molar in specimens printed at the left and right platform positions (Figure 6).

## 4. Discussion

This investigation examined how build platform location influences the dimensional accuracy of LCD-printed orthognathic surgical splints fabricated using LCD 3D printing technology. In routine digital laboratory workflows, maximizing build platform utilization while maintaining acceptable accuracy is desirable. However, evidence regarding the effect of object placement on the build platform, particularly for LCD-based printing systems, remains limited. The present findings demonstrate that printing position significantly affects precision, whereas trueness remains comparable among different positions.

The effect of three different printing positions on a medium-sized build platform was examined during the fabrication of orthognathic surgical splints using an LCD 3D printer. The results showed that surgical splints printed at the center of the build platform exhibited significantly lower RMSE values for precision compared with those printed at the left and right positions. In contrast, no statistically significant differences in trueness were observed among the three printing positions. These findings suggest that printing position primarily influences the repeatability and consistency of fabrication, rather than the overall dimensional deviation from the reference model.

Previous studies have reported that multiple factors, including printing orientation, layer thickness, material composition, and post-processing conditions, affect the accuracy of additively manufactured dental appliances. Sherman et al. indicated the printing position did not significantly influence the accuracy of study models fabricated using DLP technology, where models were placed at the center and corners of the build platform [14]. In contrast, the present study demonstrated a significant positional effect on precision when using LCD technology. This discrepancy may be attributed to differences in light projection mechanisms between DLP and LCD systems. Unlike DLP printers, which project light through optical lenses, LCD printers rely on backlight transmission through an LCD panel, potentially resulting in variations in light uniformity across the build platform. Furthermore, the rounded geometry and edge regions of the build platform may contribute to support instability and differential polymerization behavior at lateral positions. Objects printed near the periphery of the platform may be more susceptible to minor distortions during layer separation and post-processing, leading to reduced precision compared with centrally positioned objects. These observations are consistent with previous findings reporting reduced precision for objects fabricated near the edges of the build platform in vat polymerization systems [17].

Although the trueness values slightly exceeded the commonly reported clinical threshold of approximately 100–150 μm for orthognathic splints [18], the high level of precision observed across specimens suggests predictable and reproducible fabrication. In clinical practice, consistency among splints is particularly important for intraoperative positioning, where relative accuracy and repeatability can influence surgical predictability [19]. Therefore, the observed improvement in precision associated with central placement on the build platform is clinically relevant, even in the absence of significant differences in trueness.

Color map deviation analysis revealed consistent patterns of positive deviation at the incisal edges, canine cusps, premolar cusps, and mesial surfaces of the first molars across all groups (Figure 5). These areas are geometrically complex and may be more susceptible to resin accumulation or over-polymerization, particularly when printing at a 90-degree orientation. Slight negative deviations observed in certain regions may be related to variations in anti-reflective spray thickness or localized scanning artifacts. Importantly, these deviation patterns were similar across printing positions, supporting the finding that printing position did not substantially affect trueness.

### Clinical Implications

From a clinical and laboratory workflow perspective, the findings of this study suggested that preferential placement of orthognathic surgical splints at the center of the build platform may enhance fabrication precision when using LCD 3D printers. Improved precision can contribute to more predictable splint fit and positioning during orthognathic surgery, potentially reducing intraoperative adjustments and enhancing surgical efficiency. For dental laboratories utilizing LCD printing systems, strategic object placement may represent a simple and cost-effective approach to optimizing manufacturing outcomes without modifying printing parameters or materials.

Beyond orthognathic surgical splints, three-dimensional printing has been increasingly applied in various orthodontic procedures. These applications include the fabrication of study models for diagnosis and treatment planning, clear aligner models, indirect bonding trays, customized orthodontic appliances, and surgical guides for temporary anchorage devices (TADs). The dimensional accuracy and reproducibility of these printed appliances are critical for clinical success. Therefore, understanding factors that influence printing precision, such as build platform position, may also be relevant to other orthodontic applications that rely on accurate digital-to-physical transfer. Future investigations evaluating positional effects in the fabrication of those orthodontic devices may further expand the clinical significance of LCD-based additive manufacturing in orthodontics.

The main limitation of this study is that it evaluates, for an LCD 3D printer, one building platform size, a fixed printing angle, and one type of photopolymer resin. Additionally, printer-specific parameters such as light uniformity and parameter calibration could not be modified. Therefore, caution should be exercised when generalizing these findings to other LCD printers with different optical configurations or build platform dimensions. Furthermore, various types of photopolymer resins are available beyond that used in this study.

Future research should investigate the effect of build platform position across different LCD printer models, platform sizes, and printing orientations. Studies evaluating other dental applications, such as surgical guides, occlusal splints, and aligners, would further clarify the clinical relevance of printing position. In addition, combining positional analysis with variations in post-processing protocols and resin formulations may provide a more comprehensive understanding of accuracy optimization in LCD-based additive manufacturing.

## 5. Conclusions

• Printing position on the build platform significantly influenced the precision of orthognathic surgical splints fabricated using LCD 3D printing technology.
• The central region of the build platform demonstrated the lowest RMSE values for precision, indicating higher fabrication consistency.
• Trueness was not significantly affected by printing position.
• Preferential placement of surgical splints at the center of the build platform is recommended to optimize manufacturing predictability in digital orthognathic surgery workflows.

## Figures and Tables

**Table 1:** Materials, equipment, and software used in this study.

Category	Item	Manufacturer	Model/ Software Version
3D Printer	LCD 3D Printer	Ackuretta Technologies, Taiwan	SOL 130
Resin	Surgical guide resin	Ackuretta Technologies, Taiwan	CURO Guide (KT220570101)
Scanner	Optical scanner	3Shape, Denmark	E3
Post-curing unit	UV curing unit	Ackuretta Technologies, Taiwan	CURIE Plus 300
Cleaning unit	Cleaning system	Ackuretta Technologies, Taiwan	CLEANI 170
Software	Slicing software	Ackuretta Technologies	ALPHA AI Slicing V 5.1.7
Software	CAD software	Materialized (Materialise, Leuven, Belgium)	ProPlan CMF V 3.0
Software	Deviation analysis	EDF, France	CloudCompare V 2.13.2

**Table 2:** The normality test for the RMSE value of the trueness of surgical splints manufactured at different printing positions.

Tests of Normality						
RMSE of position	Kolmogorov–Smirnov ^a^			Shapiro–Wilk		
	Statistic	df	Sig.	Statistic	df	Sig.
Left	0.209	12	0.156	0.837	12	0.025
Middle	0.239	12	0.056	0.864	12	0.054
Right	0.115	12	0.200 *	0.978	12	0.976

* This is a lower bound of the true significance. ^a^. Lilliefors Significance Correction.

**Table 3:** The normality test for the RMSE value of the precision of surgical splints manufactured at different printing positions.

Tests of Normality						
RMSE of position	Kolmogorov–Smirnov ^a^			Shapiro–Wilk		
	Statistic	df	Sig.	Statistic	df	Sig.
Left	0.083	66	0.200 *	0.981	66	0.429
Middle	0.090	66	0.200 *	0.968	66	0.088
Right	0.103	66	0.080	0.970	66	0.115

* This is a lower bound of the true significance. ^a^. Lilliefors Significance Correction.

**Table 4:** The post hoc test for the RMSE value of the trueness of surgical splints manufactured at different printing positions.

Tukey HSD Multiple Comparisons of RMSE Values					
Position (I)	Position (J)	Mean Difference (I–J)	Std. Error	Sig.	95% Confidence Interval Lower Bound
Left	Middle	0.00640	0.00303	0.103	−0.0010
	Right	0.00315	0.00303	0.557	−0.0043
Middle	Left	−0.00640	0.00303	0.103	−0.0138
	Right	−0.00325	0.00303	0.537	−0.0107
Right	Left	−0.00315	0.00303	0.557	−0.0106
	Middle	0.00325	0.00303	0.537	−0.0042

**Table 5:** The post hoc test for the RMSE value of the precision of surgical splints manufactured at different printing positions.

Tukey HSD Multiple Comparisons of RMSE Values					
Position (I)	Position (J)	Mean Difference (I–J)	Std. Error	Sig.	95% Confidence Interval Lower Bound
Left	Middle	0.02548 *	0.00489	0.000	0.0139
	Right	0.00252	0.00489	0.864	−0.0090
Middle	Left	−0.02548 *	0.00489	0.000	−0.0370
	Right	−0.02296 *	0.00489	0.000	−0.0345
Right	Left	−0.00252	0.00489	0.864	−0.0141
	Middle	0.02296	0.00489	0.000	0.0114

## Data Availability

The datasets supporting the conclusions of this study are included in this article. The datasets used and analyzed in the current study are available from the corresponding author upon personal request.

## Abbreviations

The following abbreviations are used in this manuscript:

2D	Two-dimensional
3D	Three-dimensional
CBCT	Cone-beam computed tomography
DLP	Digital light processing
LCD	Liquid-crystal display
OGS	Orthognathic surgery
RMSE	Root mean square error
SLA	Stereolithography
STL	Standard tessellation language
